# 985. Practice Patterns of Antimicrobial Suppression Therapy in Total Knee Arthroplasty Infections

**DOI:** 10.1093/ofid/ofac492.827

**Published:** 2022-12-15

**Authors:** Allison Lastinger, Jacob Scribner

**Affiliations:** West Virginia University Hospital, Morgantown, West Virginia; West Virginia University Hospital, Morgantown, West Virginia

## Abstract

**Background:**

There are no current established guidelines for the role of antimicrobial suppression therapy in management of prosthetic joint infection (PJI). In 2013, the IDSA provided initial treatment guidelines for PJI but did not address suppressive therapy. The guidelines do not address suppressive therapy beyond the initial 4-6 weeks for non-staphylococcal infections nor do they address suppressive therapy after 3-6 months of treatment for Staphylococcal infections. Practitioners must delicately balance the risk of PJI recurrence and the negative effects of continued antibiotic therapy including toxicity and resistance. In this study, we surveyed Infectious Disease providers to better understand their antimicrobial suppression practice patterns in different clinical scenarios.

**Methods:**

We surveyed Infectious Disease providers across the United States from April 2022 to May 2022. We received 78 unique response from 31 different states. Basic demographic data was obtained. Providers presented six multiple choice clinical scenarios and were asked if they would start suppressive therapy and the duration of suppressive therapy.

**Results:**

The preliminary data revealed that practice patterns vary greatly amongst providers in the management of antibiotic suppressive therapy in PJI. Of the 6 scenarios, only half had greater than 50% consensus (50.6%, 51.3% and 70.1%). Scenario 1 involved a patient who underwent a single stage revision. It was of particular interest as 51.3% of providers indicated they would stop antimicrobial therapy and 48.7% indicated they would extend antimicrobial therapy with 25.6% electing for lifelong suppressive therapy.

**Conclusion:**

Practice patterns for antimicrobial suppression therapy in knee PJI varies significantly among providers. Even with IDSA guidelines addressing Staphylococcal PJI, the practice patterns among infectious diseases providers still varied greatly especially with regards to single stage revisions. Additional randomized controlled trials examining the best way to manage antimicrobial suppression in PJI would be helpful, and would allow for development of evidence-based guidelines. We also plan to survey orthopedic surgeons using the same clinical scenarios to see if there is a difference in management styles.

**Disclosures:**

**All Authors**: No reported disclosures.

